# Deep Venous Thrombosis Within a Complete Duplication of the Left Femoral Vein

**DOI:** 10.7759/cureus.63234

**Published:** 2024-06-26

**Authors:** Kaley A Coffey, Ali Kimyaghalam

**Affiliations:** 1 General Surgery, Western Reserve Health Education, Warren, USA; 2 General Surgery, Trumbull Regional Medical Center, Warren, USA

**Keywords:** xarelto, extensive thrombus, mechanical thrombectomy (mt), pulmonary embolism (pe), venogram, anticoagulation therapy, anatomical variation, venous thrombosis, femoral vein duplication, deep venous thrombosis

## Abstract

Venous duplications, particularly in the femoral vein, are rare anatomical variations that can complicate the clinical presentation and management of deep venous thrombosis (DVT). This case describes an elderly female who was diagnosed by her primary care physician with a left lower extremity DVT one week prior to her presentation and had been prescribed Xarelto. Despite strict adherence to therapy, her left leg pain, swelling, and discoloration worsened, prompting her hospital admission. On physical examination, her left leg was markedly swollen, violaceous, and tender. A repeat compression ultrasound upon admission revealed an occlusive thrombus within the left common femoral vein. Given the diagnosis of phlegmasia, cerulea dolens, the patient was at risk for irreversible venous gangrene and possible limb loss. Therefore, she was taken to the operating room for venography and a mechanical thrombectomy. Venography of the left lower extremity uncovered an extensive thrombus within a complete duplication of the left femoral vein, as well as in the left common femoral and iliac veins. Thrombosis in a duplicated femoral vein, though rare, is a significant clinical entity. This case highlights the importance of considering anatomical anomalies in patients with refractory symptoms and emphasizes the role of detailed imaging for accurate diagnosis and tailored treatment.

## Introduction

Deep venous thrombosis (DVT) is a significant medical condition characterized by the formation of a thrombus within the deep veins, most commonly in the lower extremities. This condition poses a considerable risk of complications such as pulmonary embolism and post-thrombotic syndrome. Venous duplications, although relatively rare, introduce additional complexity to the diagnosis and management of DVT. The femoral vein is one of the major venous conduits in the lower extremities, and duplications in this vein are particularly unusual, complicating the clinical picture and necessitating advanced diagnostic and therapeutic approaches [1-3].

We present the case of an 88-year-old female who was diagnosed with symptomatic DVT and treated with rivaroxaban; however, her symptoms did not improve. Approximately one week later, further evaluation with a venogram revealed extensive thrombus within the common femoral vein, external iliac vein, and throughout the entire duplicated femoral vein.

The rarity of femoral vein duplication and its impact on DVT pathophysiology necessitate a detailed examination of such cases. This case report aims to contribute to the limited literature on DVT in duplicated venous systems, discussing the diagnostic intricacies, pathophysiological considerations, and management strategies for optimal patient outcomes [4].

## Case presentation

An 88-year-old Caucasian female presented to the emergency department complaining of dark, bloody stools for two days. She reported that approximately one week ago, she was diagnosed with a left lower extremity DVT and was prescribed rivaroxaban. She endorsed worsening pain and swelling throughout her left leg. She denied any lightheadedness, chest pain, shortness of breath, or abdominal or epigastric pain. Vital signs obtained during triage were reported as a blood pressure of 148/88 mmHg (recorded low of 122/69 mmHg), a heart rate of 90 beats/min (recorded high of 97 beats/min), and a respiratory rate of 18-22 breaths per minute. She was afebrile with a room air oxygen saturation of 99%.

Her past medical history was significant for hypertension managed with metoprolol and chronic venous insufficiency, which required endovenous laser treatment approximately one year ago. On the lower extremity exam, her left leg was visibly more edematous compared to her right leg, with early phlegmasia cerula dolens in the distal foot extending to the mid-calf. She had a moderate amount of pain on palpation throughout her left leg. She had bilateral palpable femoral pulses, with dopplerable pedal signals bilaterally. The patient remained hemodynamically stable and was afebrile, saturating well on room air. Initial laboratory data was insignificant, with a hemoglobin of 12.7 g/dL, a hematocrit of 36.7%, and normal kidney function. Venous duplex studies of the left lower extremity were performed in the emergency department, which revealed an extensive left common femoral vein DVT. Given that the patient was hemodynamically stable, the decision was to address her left lower extremity DVT as soon as possible. The patient was taken for an urgent left lower extremity venogram with mechanical thrombectomy, along with an inferior vena cava filter placement, given the patient is experiencing bleeding on anticoagulation.

**Figure 1:**
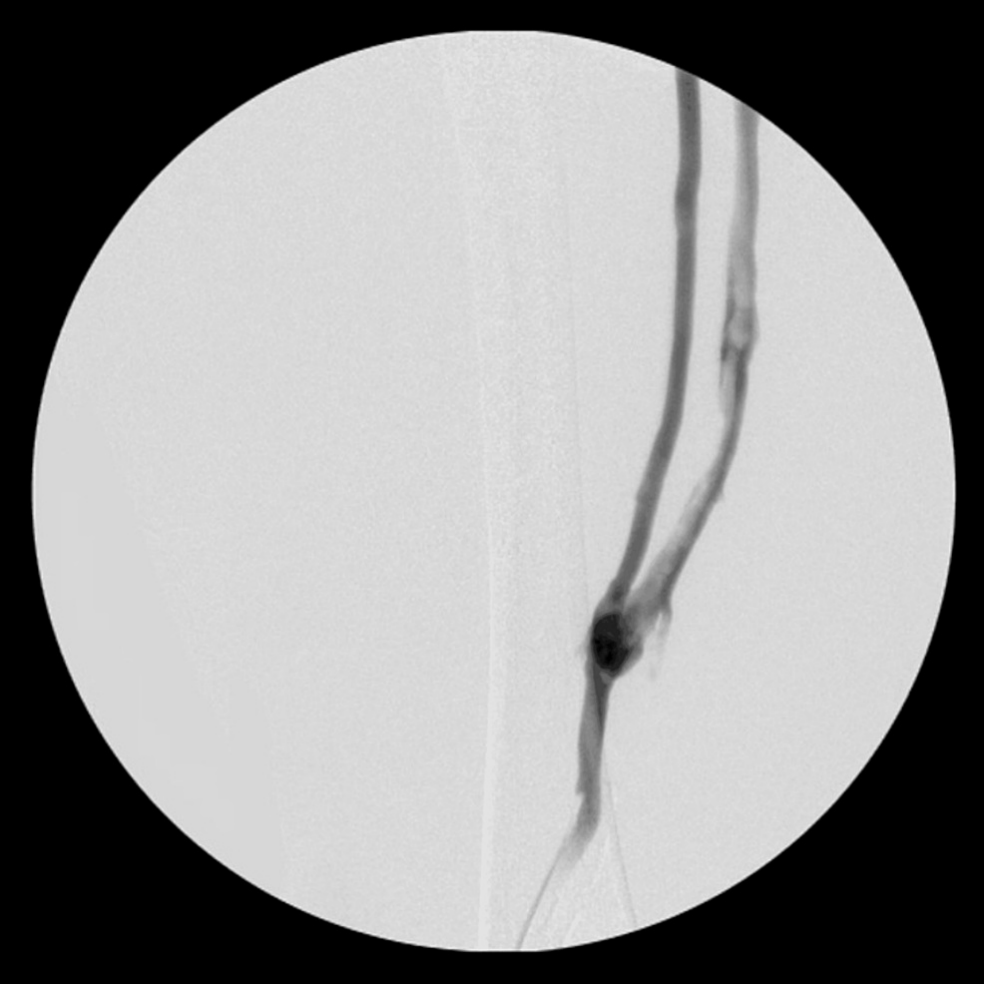
Venogram of the left lower extremity displaying the distal end of the duplicated left femoral vein

**Figure 2:**
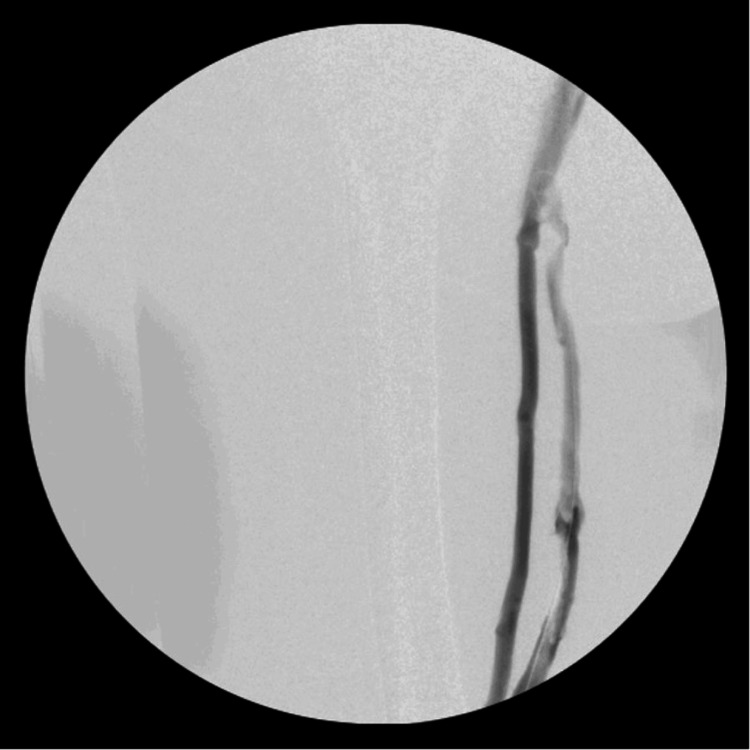
Venogram of the left lower extremity displaying the proximal end of the duplicated left femoral vein

**Figure 3:**
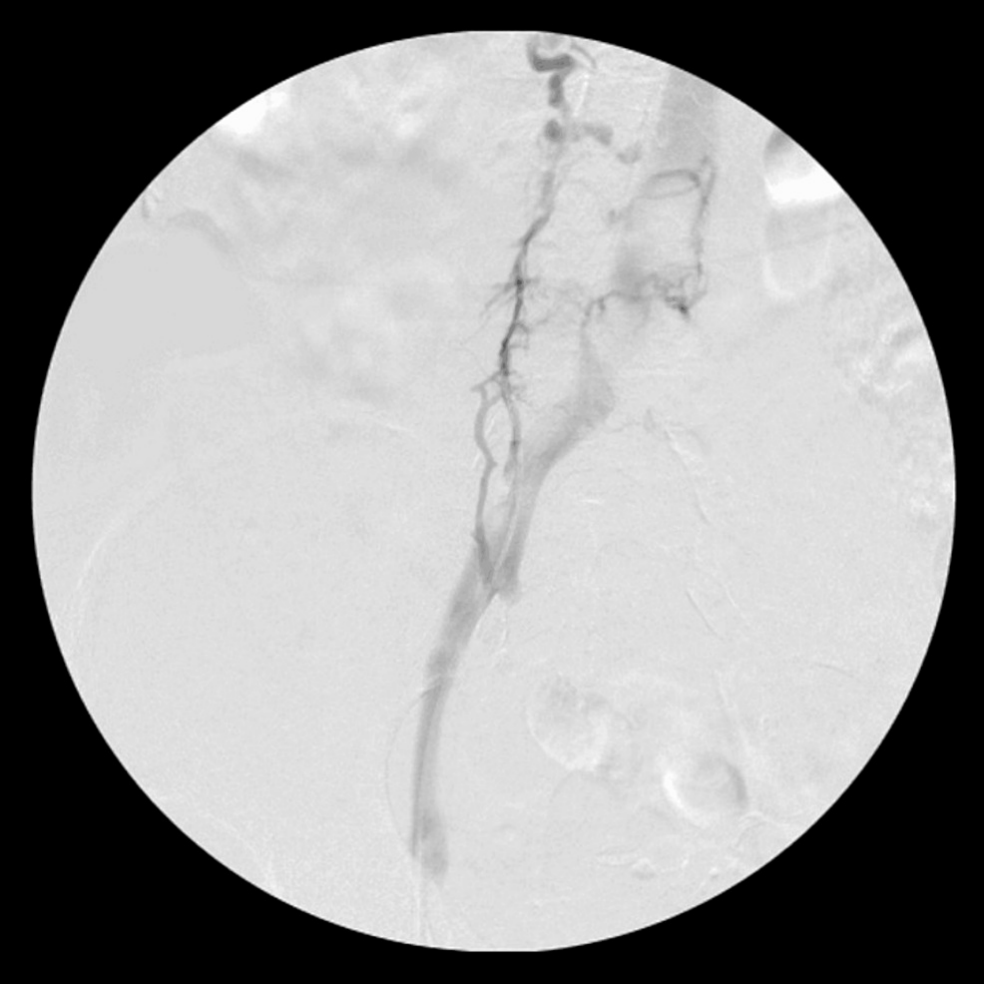
Venogram of the left lower extremity displaying extensive thrombus within the duplicated femoral vein, common femoral vein, and iliac vein

**Figure 4:**
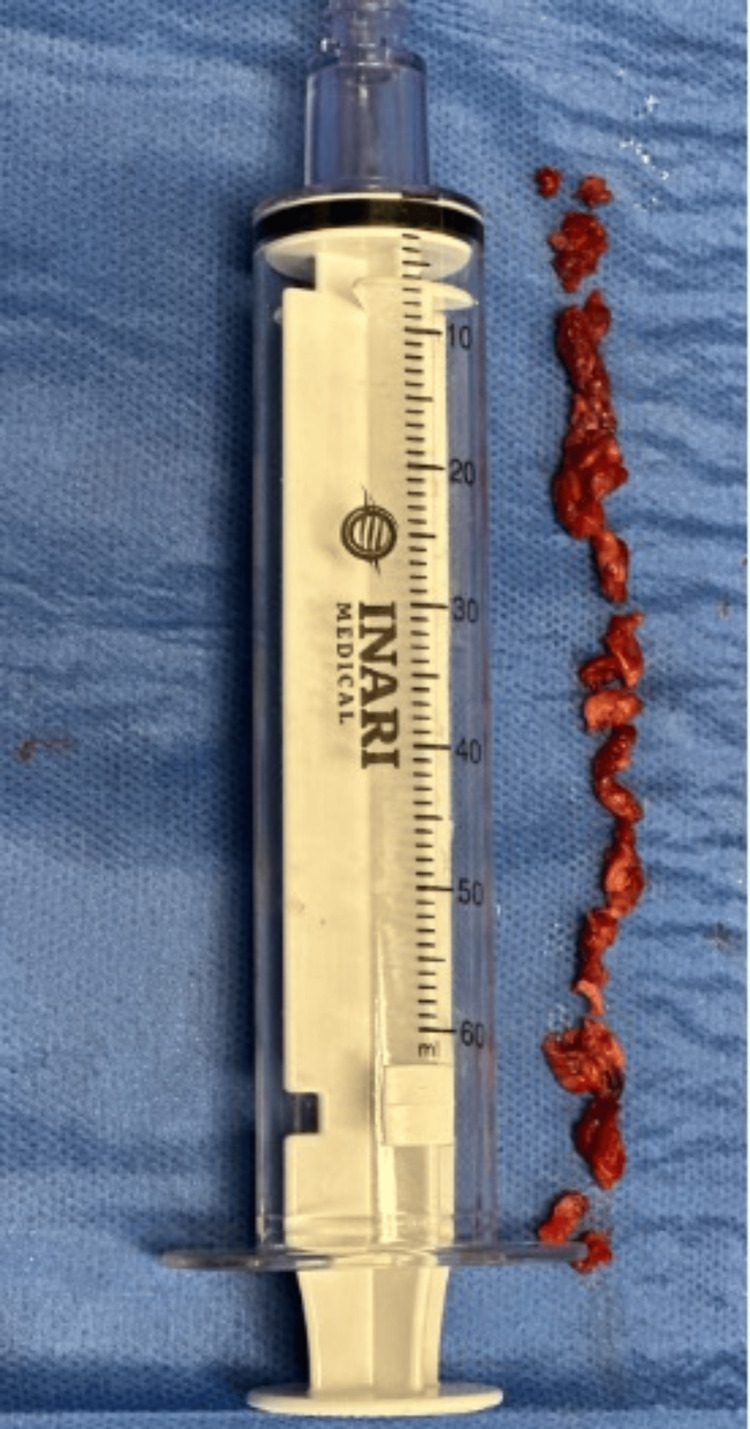
Thrombus retrieved by the FlowTriever device (Inari Medical, Inc., Irvine, CA, USA)

The patient was initially placed in the prone position, and the left popliteal vein was accessed with ultrasound guidance. Venography revealed an extensive thrombus in what appeared to be a complete duplication of the femoral vein (Figures 1-2). Thrombus was also noted within the left common femoral vein and iliac vein (Figure 3). After heparinization, a mechanical thrombectomy was performed, with three additional passes performed to improve the patency of the vein (Figure 4). The final venogram at the end of the case confirmed the resolution of the venous thrombus. The patient was then positioned supine to access the right common femoral vein for inferior vena cava filter placement.

The patient was started on 81 mg of aspirin on post-operative day one with the discontinuation of her rivaroxaban. She was able to ambulate without issue and reported a significant improvement in her pain with resolving edema. She denied any further episodes of melena and was discharged later that day. On her follow-up visit one week later, the patient reported complete resolution of her left leg pain, edema, and discoloration.

## Discussion

The occurrence of a thrombus within a duplicated femoral vein is a relatively uncommon phenomenon that presents unique diagnostic and therapeutic challenges. Venous duplication, while rare, introduces complexities in clinical presentation and management due to its potential to mask the extent of thrombus formation and alter hemodynamic flow. Asymptomatic cases are often missed during initial duplex ultrasound exams, which increases the risk of serious complications like pulmonary embolism [1].

In this case, the patient, an 88-year-old female, became symptomatic with leg edema and pain due to extensive proximal occlusion in the common femoral vein and iliac vein. If the thrombus had been confined to the duplicated femoral vein alone, it might have gone undiagnosed with a patent primary femoral vein. This scenario underscores the importance of considering venous duplication in patients with persistent symptoms despite initial treatment and highlights the increased risk of embolic events.

The incidence of femoral vein duplication is relatively low, estimated at less than 10% in the lower extremity. A study by Simpson et al. found that 10.1% of patients had at least one duplicated venous segment, with 0.4% having a thrombus within the duplicated segment. The rarity of thrombus in duplicated segments further emphasizes the uniqueness of our case, where the thrombus extended through the entire duplicated femoral vein system [1].

Research by Liu et al. on the anatomical variations predisposing to DVT revealed that duplicated femoral veins could contribute to "silent" DVTs. Their study demonstrated that multiplicity in venous structures provides internal collaterals, reducing the likelihood of complete occlusion and symptomatic presentation. In their study, multiple femoral veins were found in 31% of limbs, with a higher incidence of DVT in these limbs compared to those with single femoral veins. Notably, 41% of DVT cases in limbs with multiple veins were asymptomatic, compared to 72% in limbs with single femoral veins [2].

The pathophysiology of thrombus formation in duplicated veins involves Virchow's triad: venous stasis, endothelial injury, and hypercoagulability. Duplicated venous structures can promote venous stasis due to altered hemodynamics and flow velocities. In this patient, advanced age and comorbidities likely contributed to a hypercoagulable state, further predisposing her to thrombus formation [4].

Management of DVT in the context of venous duplication typically follows standard anticoagulation protocols but may require more aggressive or adjunctive therapies due to anatomical complexities. While the patient was initially managed with rivaroxaban, the extensive thrombosis necessitated further evaluation and consideration of additional treatments such as catheter-directed thrombolysis or mechanical thrombectomy [5].

The prognosis for patients with DVT in duplicated veins hinges on timely and accurate diagnosis and effective management. Long-term anticoagulation is essential to prevent recurrence and manage complications. Regular follow-up with duplex ultrasonography is recommended to monitor thrombus resolution and assess for complications such as post-thrombotic syndrome [6].

## Conclusions

The presence of venous duplication can complicate the clinical picture and lead to missed diagnoses if not properly evaluated. The patient’s presentation and management demonstrate the importance of detailed imaging and consideration of anatomical anomalies in patients with refractory DVT symptoms. Management primarily involves anticoagulation, with mechanical interventions reserved for select cases. A multidisciplinary approach, involving vascular specialists, radiologists, and hematologists, is essential for optimizing patient outcomes. Further research is essential to establish guidelines for the optimal management of DVTs in patients with venous duplications, ensuring that these rare but impactful conditions are effectively treated.
